# Modeling and Composition Design of Low-Alloy Steel’s Mechanical Properties Based on Neural Networks and Genetic Algorithms

**DOI:** 10.3390/ma13235316

**Published:** 2020-11-24

**Authors:** Zhenlong Zhu, Yilong Liang, Jianghe Zou

**Affiliations:** 1 College of Materials and Metallurgy, Guizhou University, Guiyang 550025, China; gs.zlzhu17@gzu.edu.cn (Z.Z.); gs.zoujh19@gzu.edu.cn (J.Z.); 2 Guizhou Key Laboratory of Materials Strength and Structure, School of Mechanical Engineering, Guizhou University, Guiyang 550025, China; 3 High Performance Metal Structure Material and Manufacture Technology National Local Joint Engineering Laboratory, School of Mechanical Engineering, Guizhou University, Guiyang 550025, China

**Keywords:** alloy steel, neural networks, genetic algorithm, mechanical properties

## Abstract

Accurately improving the mechanical properties of low-alloy steel by changing the alloying elements and heat treatment processes is of interest. There is a mutual relationship between the mechanical properties and process components, and the mechanism for this relationship is complicated. The forward selection-deep neural network and genetic algorithm (FS-DNN&GA) composition design model constructed in this paper is a combination of a neural network and genetic algorithm, where the model trained by the neural network is transferred to the genetic algorithm. The FS-DNN&GA model is trained with the American Society of Metals (ASM) Alloy Center Database to design the composition and heat treatment process of alloy steel. First, with the forward selection (FS) method, influencing factors—C, Si, Mn, Cr, quenching temperature, and tempering temperature—are screened and recombined to be the input of different mechanical performance prediction models. Second, the forward selection-deep neural network (FS-DNN) mechanical prediction model is constructed to analyze the FS-DNN model through experimental data to best predict the mechanical performance. Finally, the FS-DNN trained model is brought into the genetic algorithm to construct the FS-DNN&GA model, and the FS-DNN&GA model outputs the corresponding chemical composition and process when the mechanical performance increases or decreases. The experimental results show that the FS-DNN model has high accuracy in predicting the mechanical properties of 50 furnaces of low-alloy steel. The tensile strength mean absolute error (MAE) is 11.7 MPa, and the yield strength MAE is 13.46 MPa. According to the chemical composition and heat treatment process designed by the FS-DNN&GA model, five furnaces of Alloy1–Alloy5 low-alloy steel were smelted, and tensile tests were performed on these five low-alloy steels. The results show that the mechanical properties of the designed alloy steel are completely within the design range, providing useful guidance for the future development of new alloy steel.

## 1. Introduction

Low-alloy steel is an important metal material in economic and defense contexts. The mechanical properties of alloy steels depend on the internal organization microstructure, and the internal organization depends on the influence of important factors, such as alloy elements and process parameters [1,2,3,4,5]. The production process involves many physical and chemical reactions, and the uncertain factors in the process are difficult to determine and evaluate. Therefore, based on the appropriate processing of actual mass production data, a composition design model with sufficient accuracy and reliability is constructed to achieve a predictable tensile strength, yield strength, and other performance indicators of steel products; the model should also reasonably reveal the composition, process, and other parameters. The relationship between the mechanical properties and processing parameters has always been of concern to scientists [6,7]. Since the mechanical properties of steel are usually determined by the internal organization, which depends on the chemical composition and process parameters, it is common to study the organizational properties or to change the processing conditions to determine their impact on the properties. Kimura et al. [8] successfully designed a new type of Fe-0.4C-2Si-1Cr-1Mo (wt %) low-alloy ultra-high-strength steel. This low-alloy ultra-high-strength steel becomes a tough material with conventional high temperature treatments, and a ductile material with conventional low-temperature treatments. Zhu et al. [9] used thermodynamic and kinetics calculation methods to design the multiphase structure of phase transformation-induced plasticity (TRIP) auxiliary steel with a composition of Fe-1.5Mn-1.5Si-0.3C (wt %). To achieve a significant increase in strength and ductility, Zhang et al. [10] used the microstructure and micromechanical properties of C-Mn weld metal to study their influence on its tensile properties by finite element analysis and realized the inverse design of the mechanical properties of the weld metal. The above modeling was done to obtain metallurgical trends that reflect the evolution of mechanical properties through designing physical experiments and transforming the trends into a mathematical model based on numerical simulation and other methods. These results can reflect the evolution of steel structures during the actual hot rolling process and have a certain reliability and universality. However, as the complexity of the evolution of the organizational structure increases, the difficulty of modeling increases exponentially, causing a certain error between the calculation results of the model and the actual evolution results, and the prediction accuracy of the model is greatly restricted. In addition, the above modeling is mainly for a single steel grade, and there are certain limitations in realizing a prediction of the structure and performance of multiple steel grades.

Based on data modeling, genetic programming (GP), fuzzy regression (FR), or artificial neural networks (ANNs) can be used to explore high-dimension and complex nonlinear relationships between various factors and determine the valuable information within the data [11,12,13,14,15,16,17]. In this regard, Li et al. [10] considered the rapid modeling and plane defect prediction of a certain rolling process and used model migration strategies to develop a new flatness defect prediction model by changing the data set used for modeling. Bustillo et al. [18], based on several data mining techniques, such as multilayer perceptrons, support vector machines, and regression trees, selected the optimal data model to predict wear during the forming process of steel component thread processing. Among them, genetic programming requires prior knowledge for prediction [19], fuzzy regression depends on user-defined instructions [20], and intelligent methods, such as neural networks, have specific advantages that do not require complicated mathematical equations to explain nonlinear and multidimensional systems [21]. Therefore, neural networks are widely used in the metallurgical industry because of their outstanding modeling capabilities.

Specifically, Shen et al. [22] designed a new type of high-strength stainless steel with desirable chemical properties through a material design method combining machine learning and genetic algorithms, and verified its excellent hardness through experiments. Datta et al. [23] used neural networks and multiobjective genetic algorithms to design high-strength multiphase steels with a customized performance. John et al. [24], based on thermodynamic and kinetic models by CALPHAD that were combined with gradient algorithm and genetic algorithm multiobjective optimization, proposed an Integrated Computational Materials Engineering (ICME) method for alloy and process design for the development of d-ferrite medium-manganese steel. The approach above used machine learning and genetic algorithms to model the material design, but they only used machine learning to establish a predictive model and used genetic algorithms to obtain an optimal point in the corresponding steel. However, as the mechanical properties may increase or decrease, it is impossible to determine how the alloying elements and processes should change. Therefore, this paper proposes a neural network and genetic algorithm combined with a forward selection-deep neural network and genetic algorithm (FS-DNN&GA) model to design a composition and heat treatment process of alloy steel. The contributions of this paper can be summarized as follows:

(1) The forward selection method (FS) is constructed to screen out the main influencing factors that affect the mechanical properties, the eigenvalues selected by the FS method are used as the input of the neural network to predict the mechanical properties of alloy steel, and the FS-deep neural network (DNN) mechanical prediction model is then constructed.
(2) The FS-DNN trained model is brought into the genetic algorithm (GA) to construct the FS-DNN&GA composition design model. As the mechanical properties increase or decrease, this model outputs the corresponding alloy elements and processes.

## 2. Data Acquisition

### 2.1. Data Sources

#### 2.1.1. American Society of Metals (ASM) Database

This article uses the ASM Alloy Center Database data to train and test the FS-DNN&GA model. The ASM Alloy Center Database includes more than 10,000 data points for alloying elements, mechanical properties, physical properties, and chemical properties, all of which were collected from published papers [25,26]. This paper selects 2000 sets of complete data for training the FS-DNN&GA model. Some of the data are shown in Table 1. The chemical composition and heat treatment process are used as characteristic values, and the tensile strength and yield strength are used as predicted values (0 means air cooling, 1 means water cooling, and 2 means oil cooling, the complete data is in Appendix A).

#### 2.1.2. Low-Alloy Steel Smelting

To objectively evaluate the accuracy of the prediction model from the perspective of actual tests, steel was smelted in 35 furnaces, and heat treatments and tensile tests were performed on the steel. This experiment used vacuum arc melting equipment (model: DHL-630, Shenyang Scientific Instrument Development Center, Chinese Academy of Sciences, Shenyang, China) to smelt the low-alloy ultra-high-strength steel. The experimental process is as follows:

(1) The surface of pure iron and pig iron raw materials was mechanically polished to remove oxide scale, and low-carbon pure iron and various iron alloys were used as raw materials to prepare steel ingots according to the nominal composition.
(2) The raw materials were placed into a water-cooled copper crucible in a magnetron tungsten vacuum arc melting furnace that was evacuated below 6.8 × 10^−4^ Pa and filled with high-purity argon (99.99%). The alloy ingot was remelted many times to ensure the uniform distribution of the alloy elements.
(3) Hot forging was performed on the sample, where the starting temperature of hot forging was 1150 °C, and the final forging temperature was 850 °C.
(4) The mechanical properties of samples under different heat treatment conditions were determined.

#### 2.1.3. Tensile Testing

**Figure 1:**
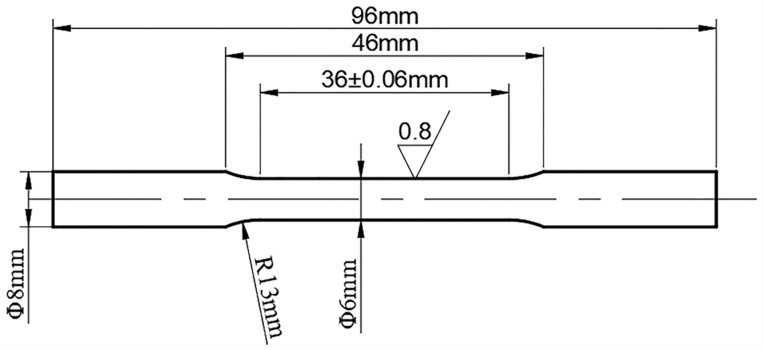
Tensile specimen.

In this experiment, a tensile specimen was prepared according to the HB5143-96 standard. The size of the specimen is shown in Figure 1. The tensile test was carried out on a universal material testing machine (model: CMT5504, MTS Industrial Systems (China) Co., Ltd. Shenzhen, China). The tensile strength and yield strength of the low-alloy ultra-high-strength steel was measured.

### 2.2. Data Preprocessing

#### 2.2.1. Data Normalization

To obtain a complete data set, the sample data for different feature dimensions was considered, and their meanings and magnitudes differed. Sample values with very large differences among the data increase the difficulty of adjusting the weight threshold during network training. To make the data less variable so that there was an excellent generalization ability and to facilitate the learning process of the neural network, the data samples were first processed in disorder and normalization. Neural networks usually use double-sigmoid functions as transfer functions, and the range of double-sigmoid functions is (0, 1), so the sample data needed to be normalized to the interval [0, 1]. Here, we used the dispersion standardization method to map the data, and the normalization formula was as follows [27]:

(1) $$X^{\prime }=\frac{\left(X-X_{\min}\right)}{\left(X_{\max}-X_{\min}\right)}$$

where *X* and *X*′ represent the values before and after normalization of the data, $X^{\prime }\in (0,1)$, respectively; $X_{\min}$ and $X_{\max}$ represent the minimum value and maximum value of the parameter in the same dimension.

#### 2.2.2. Data Outlier Elimination

To ensure the cleanliness of the sample data based on data standardization processing methods, it was also necessary to eliminate outliers in the data [28]. This is because actual production sites usually contain various sources of noise, such as system interference caused by equipment or raw materials and external noise caused by operators. The integrity and correctness of data are often affected by production interference and measurement errors. Such noise negatively affects the input samples of the predictive model. To reduce the impact, this paper uses a distance-based abnormal point detection algorithm to preprocess the sample data. The main principle is to determine whether the data point is abnormal by measuring the distance between the data tables. Each set of training samples is regarded as the utility point $d_{i}=\left\{x_{i1},x_{i2},x_{i3},\ldots ,x_{im}\right\}$, *i* = 1, 2, …, *n*. For *n* groups of m-dimensional data tables, the formula for the distance between utility points *B* and *C* is

(2) $$D_{k}\left(d_{B},d_{C}\right)={\left(\sum_{i=1}^{m}{\left|x_{Bi}-x_{Ci}\right|}^{K}\right)}^{\frac{1}{k}}$$

where *K* is any positive integer; when *K* is 1, the calculated distance is the absolute distance; when *K* is 2, the calculated distance is the Euclidean distance. Given a small positive number *δ* and an empirical critical value *N*, when $D_{k}\left(d_{B},d_{C}\right)<\delta$, utility points *B* and *C* are adjacent to each other. For any utility point, when the number of nearby points is less than *N*, the utility point is recorded as an abnormal point. This method can be used to screen the sample data to ensure its accuracy.

## 3. Method

### 3.1. FS-DNN Mechanical Prediction Model

**Figure 2:**
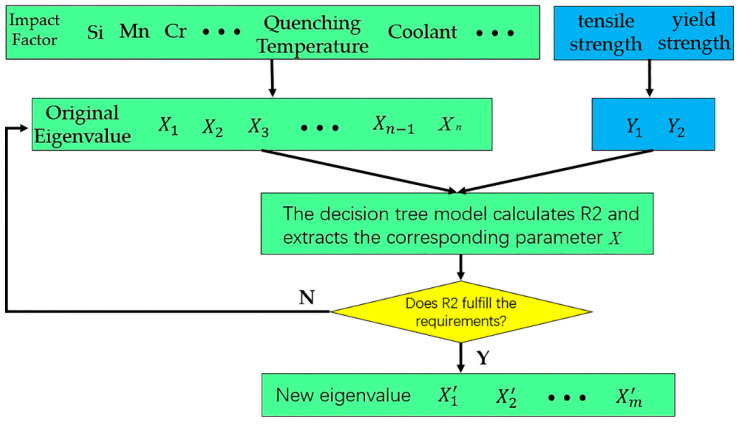
Flow chart of the forward selection (FS) method.

Three main types of factors affect the mechanical properties of alloy steel. One is alloying elements, such as C, Si, Mn, and Cr, the second is heat treatment process parameters, such as quenching temperature, tempering temperature, and coolant, and the third is plastic processing. Due to the complexity of plastic processing, if all the influencing factors are used as inputs in the prediction model, it is easy to cause overly high dimensionality, which will interfere with the calculation. Therefore, the modeling of this article does not consider plastic processing. If all alloying elements and heat treatment process parameters are used as input variables in the prediction model, the distribution of state points in the input space will be sparse, and the possibility of model overfitting will increase. Therefore, extracting important factors that impact the performance and reducing the coupling among variables can improve the quality of the input samples. This article adopts the forward selection (FS) method [29,30]. The main advantage of this method is that, after a variable is selected, the probability of the variable being highly correlated with it is reduced. Therefore, before the prediction model uses sample data for training and learning, FS can be used to determine the best combination of input variables for the prediction model. The FS method adopts a sequential selection method, and only a certain impact factor for the performance is extracted for each selection. This method is based on linear regression modeling and uses a support vector machine regression model to determine the correlation coefficient R-squared (*R*^2^) to measure the regression model under different parameters. If the value of the determination coefficient is close to 1, the correlation between the input parameter and the target value is high. A flowchart of this method is shown in Figure 2.

Step 1: Build a single-input single-output regression model for each variable given the input variables $X=\left\{X_{1},X_{2},\ldots ,X_{n}\right\}$. If the corresponding output target is *Y* and the number of input variables is n, then a single-input regression model for all influencing variables can be established: $y=f(X_{j})$,j=1,2,…,n.

Step 2: Determine the input order of the characteristic variable *X*, calculate the *R*^2^ value of all single-input regression models, select the regression model input variable corresponding to the highest *R*^2^ value, and use this variable as the first input variable of the FS model.

Step 3: Build a new multiple regression model, combine the determined variables with other remaining variables, and build m-1 new multiple regression models. The form of the new model is $y=f(X_{a},X_{j})$,j=1,2,…,a−1,a+1,…,m. Calculate the *R*^2^ value of the multiple regression model, and determine the second input variable based on the regression model with the highest *R*^2^ value.

**Figure 3:**
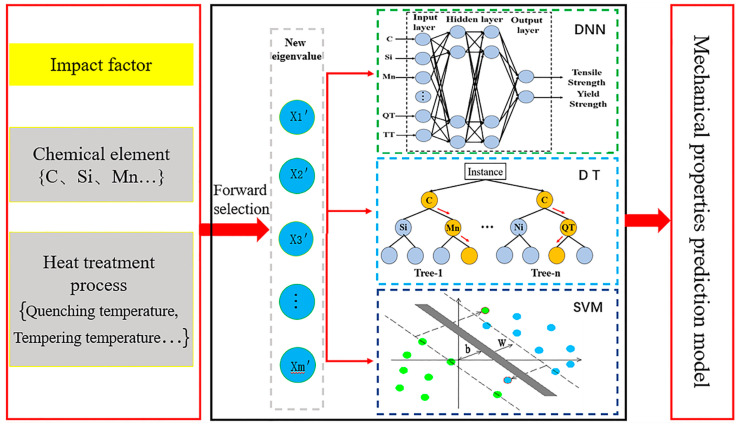
Mechanical properties prediction model.

Like the above process, the number of variables input into the FS model can be gradually increased. Usually, each new variable further increases the *R*^2^ value of the regression model. When the newly added variable is highly correlated with the selected variable, the *R*^2^ value of the regression model remains unchanged or changes very little. At this time, the FS process can be stopped. The final input variable of the FS model is the feature set of the prediction model. The new feature set is brought into the neural network and the FS-DNN mechanical prediction model is constructed. Neural network modeling is essentially a nonlinear statistical analysis technique and a black box that uses a specific set of nonlinear functions to link input data to output data. It provides a way to use an example of an objective function to find the coefficients so that a certain mapping function is as close to the objective function as possible. Srinivasu et al. [31] used the DNN method to predict the stress–strain curve of a near-beta titanium alloy, which established the best combination method to predict stress–strain curves. To prevent an algorithm from overfitting the data or coupling among the data, this paper introduces a random forest (RF) [32,33] and a support vector machine (SVM) [34] network model for comparison. The model is constructed as shown in Figure 3.

**Figure 4:**
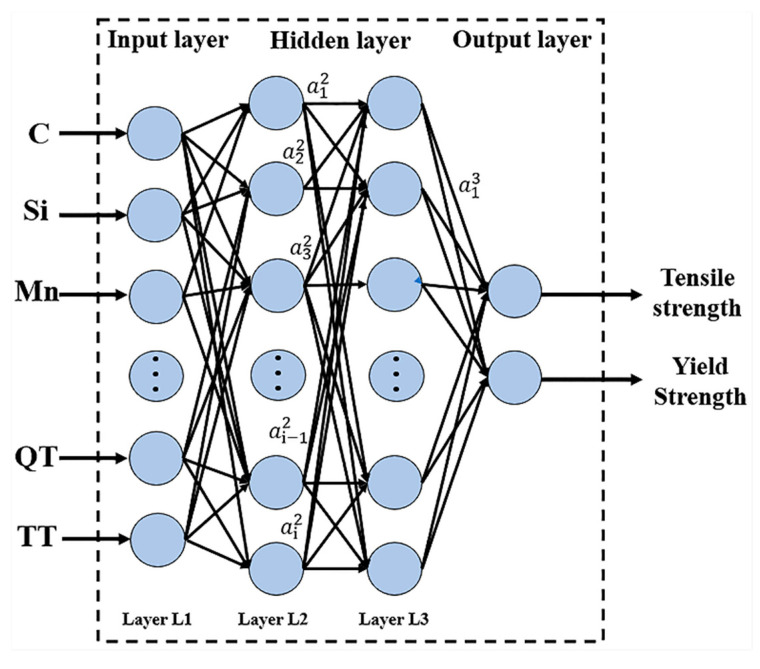
Deep neural network (DNN).

A DNN, as a technology that provides an alternative method for simulating fuzzy and complex problems, has the ability to transform nonlinear mathematical models into simplified black box structures. The advantages of using neural networks in process modeling are its learning ability, generalization ability, and nonlinearity. For the four-layer DNN in Figure 4, the eigenvalue of the DNN training sample set is recorded as $X=\left\{x_{i}\in R^{D}|i=1,2,\ldots ,n\right\}$, and the sigmoid function is used as the activation function:

(3) $$\sigma (z)=\frac{1}{1+e^{-x}}$$

The mathematical principle of forward propagation of the DNN in Figure 1 is as follows:

After inputting the feature value for the first layer, the second layer outputs 1:

(4) $$a_{1}^{2}=\sigma (z_{1}^{2})=\sigma (w_{11}^{2}x_{1}+w_{12}^{2}x_{2}+\ldots +w_{1i}^{2}x_{i}+b_{1}^{2})$$

(5) $$a_{2}^{2}=\sigma (z_{2}^{2})=\sigma (w_{21}^{2}x_{1}+w_{22}^{2}x_{2}+\ldots +w_{2i}^{2}x_{i}+b_{2}^{2})$$

(6) $$a_{i}^{2}=\sigma (z_{i}^{2})=\sigma (w_{i1}^{2}x_{1}+w_{i2}^{2}x_{2}+\ldots +w_{ii}^{2}x_{i}+b_{i}^{2})$$

The third layer output $a_{1}^{3}$ is as follows:

(7) $$a_{1}^{3}=\sigma (z_{1}^{3})=\sigma (w_{11}^{3}a_{1}^{2}+w_{12}^{3}a_{2}^{2}+\ldots +w_{1i}^{3}a_{i}^{2}+b_{1}^{3})$$

Assuming that there is a total of m neurons in the layer, the output of the l−1 neuron in the layer is $a_{j}^{l}$:

(8) $$a_{j}^{l}=\sigma (z_{j}^{l})=\sigma (\sum_{k=1}^{m}w_{jk}^{l}a_{k}^{l-1}+b_{j}^{l})$$

If l=2, then $a_{k}^{1}$ is $x_{k}$ of the input layer.

The matrix expression of the above formula can be simplified. Suppose that there are m neurons in the l−1 layer, and that there are n neurons in the l layer. The linear coefficient *w* of the l layer then forms an n*m matrix $W^{l}$, and the offset *b* of the l layer forms an n*1 vector $b^{l}$. The output a of the l−1 layer constitutes an m*1 vector $a^{l-1}$. The linear output *z* of the l layer before inactivation forms an n*1 vector $z^{l}$. The output a of the l layer constitutes an n*1 vector $a^{l}$. This is expressed by the matrix method, and the output of the l layer is

(9) $$a^{l}=\sigma (z^{l})=\sigma (W^{l}a^{l-1}+b^{l})$$

### 3.2. Constructing a Neural Network and Genetic Algorithm Component Design Model

**Figure 5:**
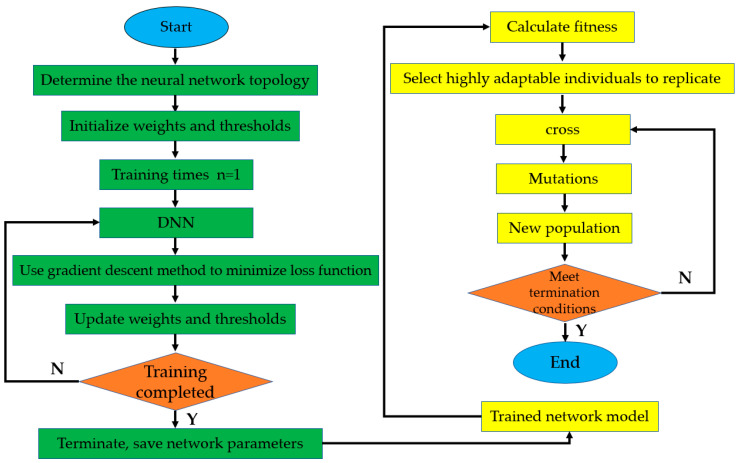
Low-alloy steel composition design model.

This paper constructs an FS-DNN&GA model to study the composition of alloy steel. First, we use the ASM Alloy Center Database data to train the FS-DNN model, save the network parameters, and bring them into the genetic algorithm. The genetic algorithm evaluates the quality of the solution through the fitness value. Therefore, the choice of its fitness value determines the search direction of the algorithm. First, the genetic algorithm is used to generate random individuals, calculate the fitness function f(x) for each individual, and call the DNN model for the calculation of the fitness function to find the best individual corresponding to f(x). A select-cross-mutate process is then started to obtain a new individual, and so on. After the iteration is completed, the individual corresponding to the best f(x) is found and the process returns. The component design process is shown in Figure 5.

## 4. Results and Discussion

### 4.1. Screening of Factors Affecting the Mechanics Based on the FS Method

In view of the screening of factors affecting the mechanical properties of steel, when the tensile strength is the research object, all the influencing parameters for the tensile strength are introduced as modeling factors. The results of the factor extraction process are shown in Table 2 (the quenching temperature is represented by QT, the tempering temperature is represented by TT, and the quenching coolant is represented by QC). It can be seen in Table 2 that, in the first step, the *R*^2^ value of the regression model built with C and the tensile strength is the largest, indicating the strongest correlation between C and the tensile strength. When taking Ni as the input parameter, other characteristic variables are added to the second step, and C, Ni, QT, and other factors are gradually selected as the input parameters of the prediction model by comparing the model’s *R*^2^ values under different variables. When Cr is added to the model in the seventh step, the change in the value of *R*^2^ is small, less than 0.5%, indicating that there is a high coupling relationship between the variable and the extracted variable. Therefore, the parameter extraction process ends.

**Figure 6:**
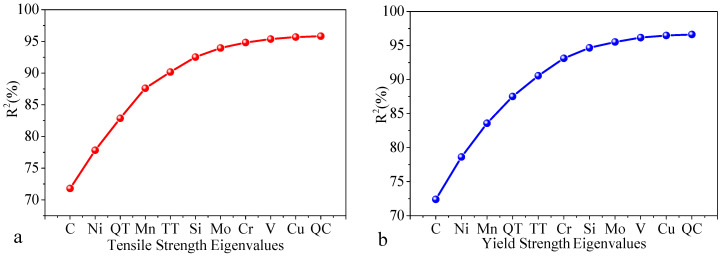
FS to determine the trends of the *R*^2^ values: (**a**) tensile strength and (**b**) yield strength.

Figure 6 shows the value of *R*^2^ in the first 11 steps according to the FS. Figure 6a shows the trend for the *R*^2^ value during the selection process of the tensile strength parameters. It can be seen in the figure that the increase in the *R*^2^ value gradually decreases and tends to flatten. This shows that, as the number of input variables increases, the influence of individual variables on the tensile strength decreases. When the *R*^2^ value reaches a certain level, the selected influencing variable has an increased degree of explanation for the tensile strength.

In the same way, the FS results of the yield strength affecting variables are shown in Figure 6b. By comparing the *R*^2^ values of the models under different variables, eight characteristic variables, such as C, Ni, and Mn, can be continuously selected as the input parameters of the yield strength prediction model. According to the graph representing the FS results, it can be seen that, in the extracted parameter set, the types of elements for the tensile strength and yield strength in the parameter sets are the same, but the order of importance of the influencing parameters is different. For the tensile strength, Mn ranks fourth in importance, while the fourth most important parameter for the yield strength is the quenching temperature. Mn ranks third, indicating that the same parameters have different effects on different properties.

To test the correlation between the extracted parameters, a heat map of the correlation between each factor was drawn, as shown in Figure 6. The value in each rectangular box indicates the degree of correlation between the factors corresponding to the horizontal and vertical coordinates. A darker color indicates a smaller correlation between the factors, and a lighter color indicates an increased correlation between the factors.

**Figure 7:**
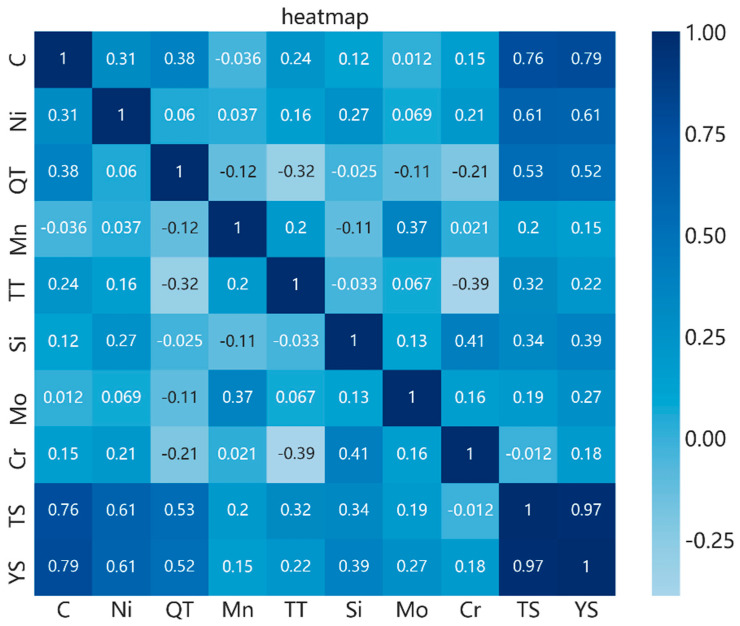
Thermal diagram of alloying elements related to tensile strength.

Figure 7 shows the following:

(1) The correlations among the eight key influencing factors selected and extracted based on the previous item are generally small, indicating that feature extraction can effectively reduce the coupling between variables and help improve the quality of input samples.
(2) Among the eight extracted parameters, the correlation between the carbon and performance is the largest, which is consistent with the results of the forward extraction process, indicating that carbon can affect the performance of steel when the composition is optimized.
(3) The correlation coefficient between the tensile strength and yield strength is 0.97, indicating a high correlation. This is because, during the design of steel products, the yield ratio (yield strength to tensile strength ratio) is usually used as the evaluation index to measure the material performance, and it is required to be within a certain parameter range. Therefore, the quality of the sample data meets certain physical metallurgy requirements.

### 4.2. Prediction of the Mechanical Properties of Low-Alloy Steel Based on the FS-DNN Model

To predict the mechanical properties of steel and provide the required information, prediction models under different properties are established to verify the predictive ability of the FS-DNN model. First, the preprocessed sample data are divided into dimensions based on the parameter extraction results from the FS and divided into different sample sets according to the main factors that influence the performance. Second, the capacity of different samples is divided. Two thousand sets of sample data are divided into a training set and a test set. Ten-fold cross-validation is used to predict the mechanical properties of alloy steel. To evaluate the effect of the regression model, we use *R*^2^ [35], mean absolute error (MAE) [36], and root mean square error (RMSE) [37] as evaluation indicators.

(10) $$R^{2}=1-\frac{\sum_{i=1}^{m}{\left(y_{i}-{\hat{y}}_{i}\right)}^{2}}{\sum_{i=1}^{m}{\left(y_{i}-{\bar{y}}_{i}\right)}^{2}}$$

(11) $$\mathrm{MAE}=\frac{1}{m}\sum_{i=1}^{m}\left|y_{i}-{\hat{y}}_{i}\right|$$

(12) $$\mathrm{RMSE}=\sqrt{\frac{1}{m}\sum_{i=1}^{m}{\left(y_{i}-{\hat{y}}_{i}\right)}^{2}}$$

Here, m is the number of samples, $y_{i}$ is the true value, ${\hat{y}}_{i}$ is the predicted value, ${\bar{y}}_{i}$ is the average value of the real labels of m samples, and i is the sample label. The evaluation results are shown in Table 3.

Based on the screening results of the important parameters from the FS, eight key feature values, such as C, Ni, and Mn, are used as inputs in the prediction model, and the output performance indicators are the tensile strength and yield strength. The comparison models are constructed as follows:

(1) The prediction model after eigenvalue screening is improved by directly introducing the eigenvalue model at the accuracy level, and the FS-DNN model has the best prediction effect. In addition, the prediction model does not need to manually set the structural parameters, which improves the stability and reliability of the prediction model and is convenient for practical application.
(2) In addition to the large improvement in the prediction accuracy of modeling after feature value screening, the feature dimensions are also reduced from the original 11 dimensions to eight dimensions, which not only simplifies the model input structure and improves the modeling speed but also reduces the dependence on prior knowledge in parameter selection.

**Figure 8:**
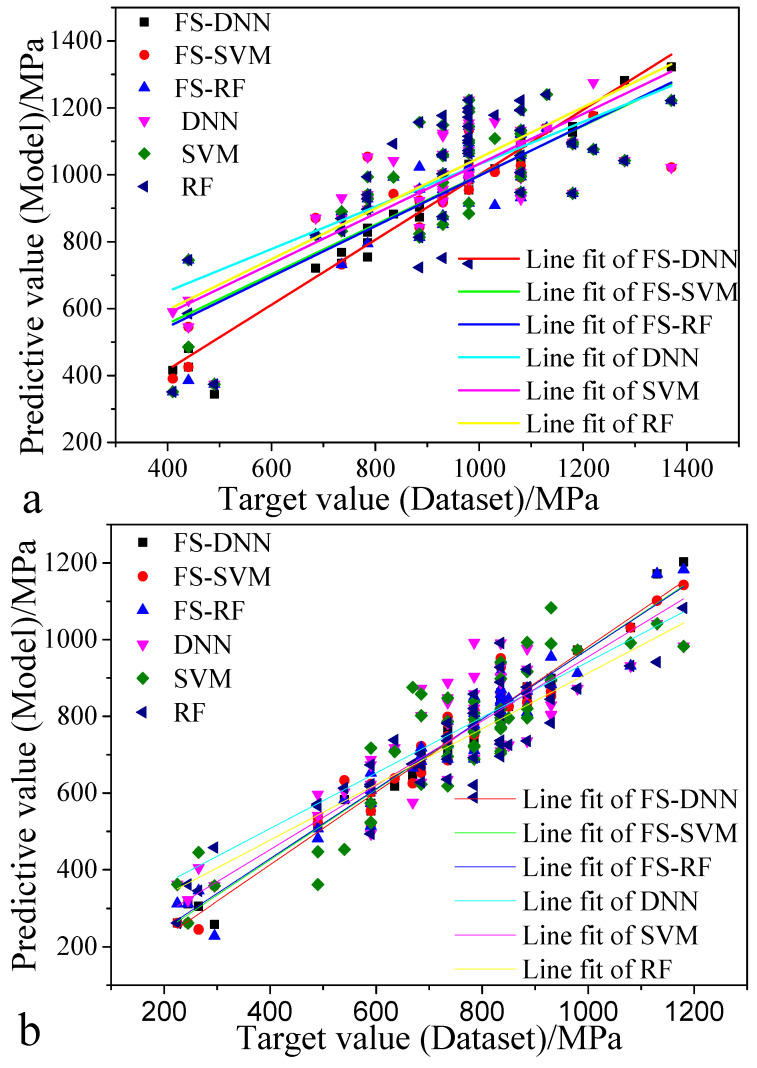
Actual values of the mechanical properties and the predicted values fit to a straight line: (**a**) tensile strength and (**b**) yield strength.

To further verify the prediction model and the effectiveness of the extraction of important parameters, the trained model is used to predict the 50 furnaces of steel smelted in the steel plant. These steels are quenched and tempered at high temperatures, and tensile specimens are made and tested on a universal testing machine. The 50 sets of data are input into the prediction model, and the measured values of the tensile strength and yield strength are compared with the predicted values and fit to a straight line, as shown in Figure 8. It can be seen in the figure that the fit of the prediction model filtered by the eigenvalues is better than that of the original model, and the FS-DNN has the best fit compared to that of the other models.

### 4.3. Composition Design of Low-Alloy Steel Based on the FS-DNN&GA Model

At present, improving the mechanical properties of alloy steels usually entails the use of metallurgical mechanisms to establish empirical formulas. However, metallurgical mechanism modeling is not only complicated but also has a single research object, which cannot meet the requirement of improving the performance of multiple steels at the same time. At the same time, physical metallurgy experiments are time consuming and not economical. In this paper, through the design of neural networks and genetic algorithms, using the mutual transformation of genotypes and phenotypes, the complex relationships among the composition, process parameters, and mechanical properties are explored, and a composition design model is established. The advantage of this model is that, when the mechanical properties are increased or decreased, the changes in the chemical composition and heat treatment process can be accurately given, and the uncertainty caused by the trial-and-error method of empirical formulas is avoided.

Through the FS method, it can be seen that the main influencing factors that affect the tensile strength and yield strength are the eight main influencing factors, such as C, Ni, and Mn. The alloy steels containing these six elements and the heat treatment process are shown in Table 4, in which we can see that the tensile strength of 18CrNiMnMoA is 1180 MPa, which is relatively low. Therefore, this paper uses the FS-DNN&GA model to increase 150 MPa sequentially on the basis of 1180 MPa and thus obtain the corresponding chemical composition and heat treatment process.

The FS-DNN&GA model is constructed to model the chemical composition and heat treatment process of low-alloy steel. Table 5 is the input boundary of the characteristic value of the FC-DNN&GA model. However, the boundary of the chemical composition and heat treatment process is determined according to the effective sample value interval of each single factor variable in the ASM Database. For example, the sample range of element C is [0.1, 0.9], because the minimum value of the low-alloy steel’s C content in the ASM Database is greater than 0.1, and the maximum value is less than 0.6. Therefore, we use the range of [0.1, 0.9] as the effective input interval. The quenching sample range is [850, 1100]. In the quenching stage, in order to make the steel fully austenitized, the lowest temperature is selected to be 850 °C. If the quenching temperature is too high, it will cause overburning of the quenched steel, causing the steel to easily crack and brittle. The toughness is insufficient, so the highest quenching temperature is 1100 °C. The tempering sample range is [150, 650]. If the tempering temperature is too low, more austenite remains, and a tempering temperature that is too high causes a phase change. Therefore, the tempering temperature boundary is set at 150 to 650 °C. Table 6 shows that the FS-DNN&GA prediction model outputs the chemical composition and heat treatment process of the tensile strength of Alloy1~Alloy5 within the boundary range of Table 5. For example, to obtain the tensile strength of Alloy1 TS [1300, 1350] MPa, the FS-DNN&GA outputs the following predictions, according to the boundary range of Table 5: C is 0.25, Si is 0.36, Mn is 0.21, Cr is 0.26, Mo is 0.21, Ni is 0.75, QT is 863, and TT is 234.

### 4.4. Composition Analysis of Newly Designed Alloy Steel

The important influencing factors of the mechanical properties of alloy steels are explored to determine whether the parameters that influence the model output can reflect the regularity of the original production data. After feature extraction, the main influencing alloy elements include C, Si, Mn, Cr, Ni, and Mo. Next, the controlled variable method is used to analyze the tensile properties of the above six single factors. The specific operations are as follows.

(1) Determine the effective sample value interval of each single factor variable and use the uniform distribution method to regenerate 20 sets of value points in this interval. For example, the sample range of element Mn is [0.1, 2.6], but, based on the one-point graph, the sample distribution is continuous and relatively concentrated in the interval [0.40, 1.9]. Therefore, the latter serves as the effective sample value interval of the element Mn.
(2) Fix the values of other parameter variables. Based on the known sample data, use valid statistical indicators (for example, mean, median, and mode) as the fixed value points of the remaining variables. In this article, the mean is used.

**Figure 9:**
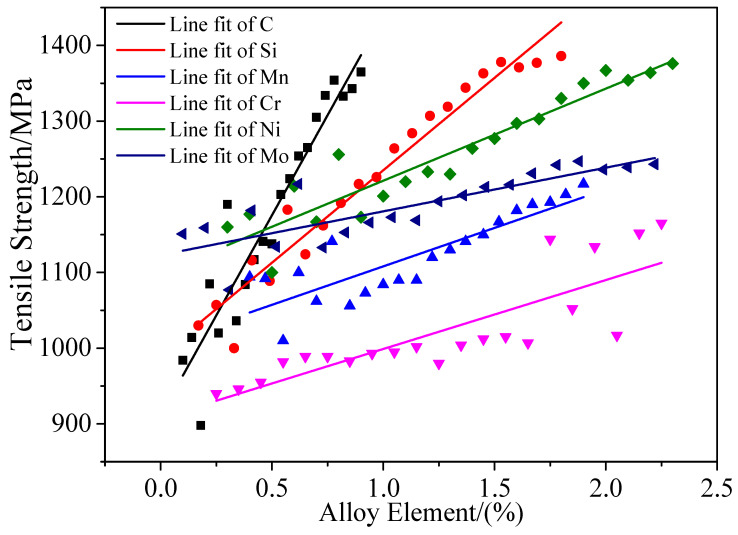
Influence of model input variables on the tensile strength.

The new sample points generated by the above process are used as the input of the prediction model, and the tensile strength values under different factors are output. The influence of the main factors on the tensile strength of steel is shown in Figure 9.

Figure 9 shows the following trends.

(1) Carbon: It can be seen in the figure that the C content and the tensile strength are directly proportional. As the carbon content gradually increases, the strength of the steel continues to increase, and the correlation is greatest. Because the strength of low-alloy ultra-high-strength steel is mainly achieved by solid solution strengthening with carbon [38], the addition of alloying elements can adjust the carbon content in the steel, and specific alloying elements, such as Ti, Nb, V, and Mo, can achieve coexistence with C in the martensite structure and achieve precipitation strengthening during tempering. However, the strength, plasticity, and initial properties contradict each other. A high strength causes a decrease in the plastic toughness, which gradually decreases. Therefore, in this model, the input C content sample interval is [0.1, 0.9] to avoid an excessive C content and poor plasticity, because materials with these properties cannot be used in actual production.
(2) Silicon: It can be seen in the figure that the Si content is proportional to the tensile strength because the addition of Si can significantly increase the tensile strength after solid solution and improve the impact toughness of the steel, mainly due to Si in the grain boundaries. Precipitation increases the activity of C and N, so it can replace the active atoms in the grain boundary regions [39] and increase the strength of steel through solid solution strengthening [40]. Even though the addition of a small amount of Si still has unexpected effects of increasing the strength of the steel and improving the impact toughness [41], it can also increase the temperature of low-temperature tempering brittleness and promote the use of fine MC particles in low-activity martensitic steel. Precipitation has the effect of precipitation strengthening [42,43]. However, too much Si leads to a decrease in toughness and welding performance [44]. Therefore, the sample interval of the input Si content in this model is [0.17, 1.8]. It can also be seen from the figure that, when the Si content exceeds 1.5%, the tensile strength tends to be flat.
(3) Manganese: It can be seen in the figure that, with the increase in Mn content, the tensile strength also increases, but this increase is relatively slow. This is because the addition of Mn can promote the formation of hardening phases, such as bainite and martensite [45,46], and inhibit the formation of ferrite-pearlite and acicular ferrite during the hardening process, thereby increasing the hardening of steel. In particular, when a small amount of N, V, or Mn is added to the steel, due to the precipitation strengthening effect of the second phase of VN [47,48], the effect of increasing the hardenability is obvious, and the relative tensile strength is not significantly improved. Thus, Mn is a microalloying element added to steel to improve its overall performance. However, an excessively high amount of Mn leads to severe segregation in the cast slab, which in turn causes the formation of banded structures during the rolling process and reduces the toughness of the steel. Therefore, the sample interval of the input Si content in this model is [0.4, 1.9].
(4) Chromium: It can be seen in the figure that the Cr content is [0.25, 2.25], and the tensile strength increases with an increase in Cr content, but it is relatively slow. This is because Cr can inhibit the growth of M_3_C during the heat treatment process, increase the tempering stability of steel, reduce carbon activity, and slow the decarburization tendency of steel. At the same time, the addition of Cr can promote the formation of bainite and martensite complex structures, which can improve the strength, toughness, and oxidation resistance of steel [49,50,51,52,53,54,55]. However, after the Cr content exceeds 1%, the tensile strength may decrease or increase, which is not stable. Zhang [49] studied the effect of Cr content on oxide dispersion strengthened (ODS) ferritic steels and found that Cr and C form carbides, which can offset the solid solution strengthening effect of a single C element, and found that a large amount of Cr-rich precipitates. However, at relatively high temperatures, certain specific elements in steel (such as W and Ti) can also induce the precipitation of Cr-rich phases [56,57]. It is well known that Cr-rich precipitates are hard and brittle. An excessive amount of Cr-rich precipitation can damage the toughness and uniformity of steel and cause microcracks. Therefore, a high Cr content may reduce the tensile strength.
(5) Nickel: It can be seen in the figure that the content of Ni is proportional to the tensile strength. Because the addition of Ni can improve the strength and ductility of steel at the same time, the improvement in the low-temperature impact performance is very obvious. An appropriate amount of Ni is added to ultra-fine WC-10Co steel [58,59,60,61,62]. Due to solid solution strengthening and WC grain refinement, the transverse strength of the steel increases significantly, but the hardness of the hard metal decreases. The addition of Ni promotes the formation of rod-like or needle-like ferrite, inhibits the precipitation of pearlite, and prolongs the precipitation time of various morphological meso-temperature transition structures. Therefore, the model is constructed with an Ni content sample interval of [0.3, 2.3].
(6) Molybdenum: Mo increases the strength by increasing the hardenability of steel. The addition of Mo can inhibit the formation of eutectoid ferrite and pearlite during the hardening process [63,64], forming a small amount of overaged martensite islands and promoting the formation of hardened phases, such as bainite and martensite [64,65], to increase the steel yield strength. Mo is a strong carbide-forming element that is mainly present in solid solutions and carbides in alloy steel. It has the effects of solid solution strengthening and precipitation strengthening and can simultaneously improve the hardenability of steel [66]. Chen et al. [67] also pointed out that adding a certain amount of Mo to NbX80 steel can significantly improve the toughness and especially the strength. Mo can also increase the AC3 temperature of the steel. Therefore, the model is constructed with a Mo content sample interval of [0.1, 2.2].

To effectively improve the mechanical properties of products, additional attention should be paid to the optimal design of components for product quality control or new product designs. Analysis of the main parameters that impact the performance of steel shows that the relationship between the output variables and the input variables based on the built FS-DNN model conforms to the metallurgical mechanisms. This shows that the built model not only can achieve high prediction accuracy but also has high reliability at the level of model regularity. Therefore, a comparison of Figure 8 and Table 6 was done to analyze whether the FS-DNN&GA model design for Alloy1~Alloy5 conforms to the metallurgical mechanisms.

(1) The alloying element parameters, including C, Si, and Ni, have a great influence on the tensile strength. It can be seen in Table 6 that, for Alloy1~Alloy5, the contents of C, Si, and Ni account for the high chemical composition of each alloy steel. At the same time, as the tensile strength increases, the content of C, Si, and Ni also increases accordingly.
(2) It can be seen in Figure 8 that the Cr content is in the range 0.25%~7.5%. As the Cr content increases, the tensile strength increases, albeit relatively slowly, and the Cr content exceeds 1.2%. With an increase in Cr content, the tensile strength is unstable. The low-alloy steel Cr designed in Table 6 increases with increasing tensile strength, and the content is in the range 0.26%~0.45%, which conforms to the metallurgical mechanisms.
(3) It can be seen in Figure 8 that the tensile strength is more sensitive to Mo than the other alloying elements within the range 0.1%~0.5%. In Table 6, with an increase in the tensile strength, the content of Mo changes from 0.21% to 0.37%.

**Figure 10:**
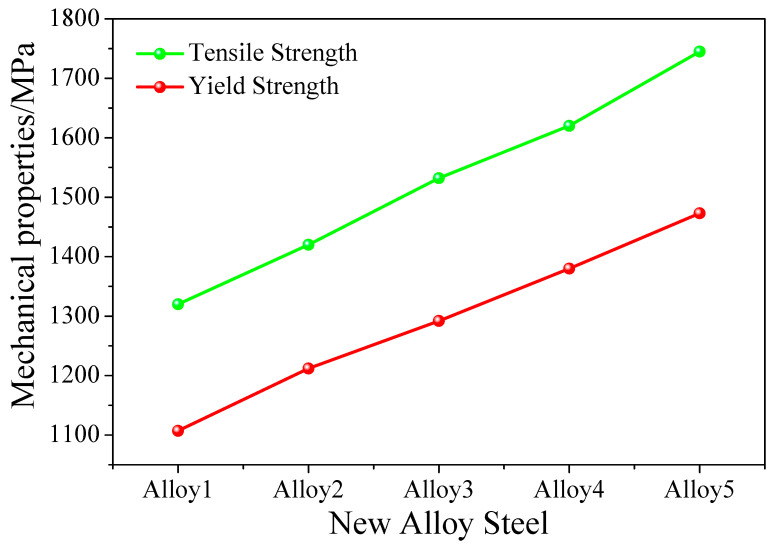
Tensile strengths and yield strengths of Alloy1~Alloy5.

To verify the accuracy of obtaining the five alloy steels, Alloy1 through Alloy5 were smelted according to the chemical composition and heat treatment process in Table 6. The alloys were processed into standard tensile specimens, and the tensile strengths and yield strengths were obtained, as shown in Figure 10. The alloy we designed fully meets the tensile strength requirements and provides guidance for future production.

In this article, 20CrMnTi is used as an example. The alloying elements of 20CrMnTi are C, Si, Mn, Cr, P, S, Ni, Cu, and Ti. Based on the forward selection method, only C, Si, Mn, Cr, and Ti in the ASM Database were retained for FS-DNN&GA model training. P, S, Ni, Cu, and other interfering alloying elements were removed. The 20CrMnTi inputs in the FS-DNN&GA model are shown in Table 7 (QT: quenching temperature; TT: tempering temperature). 20CrMnTi 0 represents the chemical composition and heat treatment process of the database. The tensile strength is 1097 MPa, which is increased by 100 MPa on the basis of 20CrMnTi 0. In the FS-DNN&GA model, the 20CrMnTi 1 tensile strength output range is [1200, 1250], and the 20CrMnTi 2 tensile strength output interval is [1300, 1350]. The FS-DNN&GA model output chemical composition and heat treatment process are shown in Table 8.

**Figure 11:**
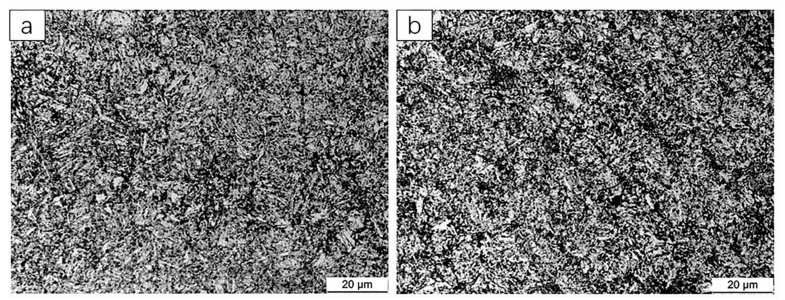
(**a**) 20CrMnTi 1 microstructure and (**b**) 20CrMnTi 2 microstructure.

Smelting was performed according to the chemical composition and heat treatment process specified in Table 6. Standard tensile specimens were processed for tensile testing. The tensile strengths of 20CrMnTi 1 and 20CrMnTi 2 were 1220 and 1346 MPa, respectively, which meet the material design requirements. Figure 11 shows 20CrMnTi 1 and the microstructure of 20CrMnTi 2. The tensile strength of Alloy2 was increased by 300 MPa to a total of 1420 MPa to obtain Alloy5. Low-alloy steels 20CrMnTi 1 and 20CrMnTi 2, compared with the 20CrMnTi 0 steels, changed little in C, Si, and Ti, but Mn and Cr highly increased. The addition of Mn can promote the formation of hardening phases; for example, bainite and martensite [32,33] can significantly increase the tensile strength and improve the impact toughness of steel. The addition of Cr promotes the formation of rod-shaped or acicular ferrite and can simultaneously improve the strength and ductility of the steel. The quenching temperatures of low-alloy steel 20CrMnTi 1 and 20CrMnTi 2 were higher than that of 20CrMnTi 0, and the ferrite in the structure after quenching was no longer in block shape, but rather in a flake shape and an island shape. At this time, the presence of ferrite had the same effect on the tensile strength and the yield strength of the steel. As the quenching temperature increased, the amount of ferrite contained in the steel decreased, so the tensile strength increased. 20CrMnTi 1 and 20CrMnTi 2 were subject to low-temperature tempering. As the low-temperature tempering temperature increased, the internal atomic mobility increased. The supersaturated carbon in the martensite began to gradually precipitate in the form of carbides. Regarding the carbon in the martensite, the degree of supersaturation was continuously reduced, and the diffusion of carbon atoms caused the content of carbon in the surrounding ferrite to gradually increase. The matrix was still dominated by martensite. Therefore, the tensile strength of the sample increased.

Aiming at the mechanical properties and composition design of low-alloy steel, based on the perspective of data modeling, this paper conducts modeling research based on neural network mechanical properties and composition design, and proposes a combination of a forward selection neural network and genetic algorithm (FS-DNN&GA). The model meets the design requirements of the mechanical properties. This model can also be used to elongate or reduce the area of low-alloy steel to quickly respond to market demand and improve the quality of product design.

## 5. Conclusions

Considering the high-dimensional issues that may be caused by the direct introduction of parameter modeling, screening important factors, such as the entry point, and using the FS method to extract important factors reduce the coupling between variables and simplify the input of the prediction model. Introducing the FS method effectively improved the generalization ability and efficiency of the alloy design. The FS-DNN mechanical prediction model is constructed by combining it with a neural network. The FS-DNN model has the best predictive effect on mechanical properties. Among them, the tensile strength R2, MAE, and RMSE are 0.953, 14.736 MPa, and 23.993 MPa, respectively, and the yield strength R2, MAE, and RMSE are, respectively, 0.962, 13.46 MPa, and 20.31 MPa. Experiments have shown that the model can effectively improve the prediction accuracy while simplifying the input structure. The FS-DNN&GA component design model is constructed based on the neural network prediction model FS-DNN and GA. By changing the model input variable range, the output performance index change trend was explored, and the influence of various factors on the mechanical properties is explained by the relationship with the metallurgical mechanisms. Experimental verification shows that the tensile strengths of low-alloy steels Alloy1~Alloy5 are 1320, 1420, 1532, 1620, and 1745 MPa, respectively, which are completely within the design interval of the FS-DNN&GA model. The FS-DNN&GA composition design model can be used for future metallurgical development.

## Figures and Tables

**Table 1:** Partial data of the American Society of Metals (ASM) Alloy Center Database.

Number	Grade	Chemical Composition (%)			Quenched		Tempering		σ_b_/MPa	σ_s_/MPa
		C	…	Cr	Temperature	Coolant	Temperature	Coolant		
1	15Cr	0.15	…	0.85	880	0	200	0	390	195
2	12CrMo	0.12	…	0.55	900	2	650	2	440	275
3	20CrMnTi	0.20	…	1.15	890	1	230	2	615	395
4	40CrMnMo	0.40	…	1.05	860	1	460	1	835	640
5	20CrMnMoB	0.20	…	1.65	850	1	500	2	880	735
6	30CrMnSi	0.30	…	0.95	860	0	200	2	685	460
7	35CrMoV	0.35	…	1.15	860	0	400	2	880	745
8	34CrNi3Mo	0.34	…	0.90	860	1	460	1	765	636
9	30Cr2Ni2Mo	0.30	…	2	870	1	500	2	900	700
10	24Cr2Ni4MoV	0.24	…	1.65	860	0	200	2	1000	870

**Table 2:** Results of FS of parameters that impact the tensile strength.

The Value of Coefficient of Determination *R*^2^ (%) from Step 1 to Step 12											
	1	2	3	4	5	6	7	8	9	10	11
C	71.79										
Ni	63.56	77.82									
QT	64.26	73.66	82.87								
Mn	63.47	72.85	81.24	87.61							
TT	47.84	75.46	80.57	85.47	90.16						
Si	55.53	71.32	81.02	85.32	88.65	92.25					
Mo	41.5	70.54	79.35	85.54	88.32	92.45	93.96				
Cr	21.57	70.32	79.56	85.68	88.12	92.11	93.35	94.82			
V	15.76	69.23	79.12	85.12	88.07	91.97	93.09	94.3	95.16		
Cu	12.2	69.23	78.32	84.87	88.17	92.07	92.94	94.22	94.38	95.28	
QC	23.73	69.17	78.15	84.56	88.01	92.18	93	94.12	94.42	94.52	95.22

**Table 3:** Evaluation indexes of the mechanical prediction model.

Mechanical Properties	Evaluation Index	Modeling after Eigenvalue Screening			Introduce All Eigenvalue Modeling		
		DNN	SVM	RF	DNN	SVM	RF
TS	*R* ^2^	0.953	0.935	0.921	0.752	0.802	0.673
	MAE	14.736	16.75	17.19	23.95	21.69	26.02
	RMSE	23.993	24.247	25.35	31.34	29.78	33.46
	Eigenvalues	8	8	8	11	11	11
YS	*R* ^2^	0.962	0.942	0.938	0.752	0.765	0.673
	MAE	13.46	14.25	14.04	20.95	21.39	18.12
	RMSE	20.31	21.57	22.25	28.33	27.78	26.46
	Eigenvalues	8	8	8	11	11	11

**Table 4:** 18CrNiMnMoA chemical composition.

Composition and Processing	C	Si	Mn	Cr	Mo	Ni	QT	TT	TS
18CrNiMnMoA	0.18	0.27	0.85	1.15	0.25	1.15	830	200	1180

**Table 5:** Composition design input range.

Composition and Processing	C	Si	Mn	Cr	Mo	Ni	QT	TT
Min	0.10	0.17	0.40	0.25	0.10	0.30	850	200
Max	0.90	1.80	1.90	2.25	2.20	2.30	950	650

**Table 6:** Characteristic elements corresponding to the tensile strength output range.

Composition and Processing	Min	Max	C	Si	Mn	Cr	Mo	Ni	QT	TT
Alloy1 TS	1300	1350	0.25	0.36	1.19	0.26	0.21	0.75	863	234
Alloy2 TS	1400	1450	0.32	0.55	1.28	0.27	0.25	1.16	918	358
Alloy3 TS	1500	1550	0.38	1.02	1.43	0.23	0.32	1.45	882	454
Alloy4 TS	1600	1650	0.43	1.6	1.19	0.38	0.36	1.65	869	320
Alloy5 TS	1700	1750	0.45	1.8	1.23	0.45	0.37	2.25	864	469

**Table 7:** Composition design input range.

Composition and Processing	C	Si	Mn	Cr	Ti	QT	TT
Min	0.17	0.17	0.80	1.00	0.04	850	200
Max	0.23	0.37	1.10	2.25	0.1	950	200
20CrMnTi 0	0.20	0.22	0.89	1.04	0.065	880	200

**Table 8:** Characteristic elements corresponding to the tensile strength output range.

Composition and Processing	Min	Max	C	Si	Mn	Cr	Ti	QT	TT
20CrMnTi 1	1200	1250	0.21	0.23	0.92	1.06	0.064	886	218
20CrMnTi 2	1300	1350	0.23	0.24	0.96	1.15	0.070	892	232

## Appendix A

**Table A1:** Partial data of the ASM Alloy Center Database. (0 means air cooling, 1 means water cooling, and 2 means oil cooling).

Number	Grade	Chemical Composition (%)												Heat Treatment Process				Mechanical Properties	
		C	Si	Mn	Cr	Mo	Ni	W	Ti	V	Cu	p	S	Quenched Temperature	Coolant	Tempering Temperature	Coolant	σ_b_/MPa	σ_s_/MPa
1	20Mn2	0.205	0.27	1.6	0	0	0	0	0	0	0	0	0	880	1, 2	440	0, 1	785	590
2	30Mn2	0.305	0.27	1.6	0	0	0	0	0	0	0	0	0	840	1	500	1	785	635
3	35Mn2	0.355	0.27	1.6	0	0	0	0	0	0	0	0	0	840	1	500	1	835	685
4	40Mn2	0.405	0.27	1.6	0	0	0	0	0	0	0	0	0	840	1, 2	540	1	885	735
5	45Mn2	0.455	0.27	1.6	0	0	0	0	0	0	0	0	0	840	2	550	1, 2	885	735
6	50Mn2	0.51	0.27	1.6	0	0	0	0	0	0	0	0	0	820	2	550	1, 2	930	785
7	20MnV	0.205	0.27	1.45	0	0	0	0	0	0.095	0	0	0	880	1, 2	200	0, 1	785	590
8	27SiMn	0.28	1.25	1.25	0	0	0	0	0	0	0	0	0	920	1	450	1, 2	980	835
9	35SiMn	0.36	1.25	1.25	0	0	0	0	0	0	0	0	0	900	1	570	1, 2	885	735
10	42SiMn	0.42	1.25	1.25	0	0	0	0	0	0	0	0	0	880	1	590	1	885	735
11	20SiMn2MoV	0.2	1.05	2.4	0	0.35	0	0	0	0.085	0	0	0	900	2	200	0, 1	1380	0
12	25SiMn2MoV	0.25	1.05	2.4	0	0.35	0	0	0	0.085	0	0	0	900	2	200	1, 2	1470	0
13	37SiMn2MoV	0.36	0.75	1.75	0	0.45	0	0	0	0.085	0	0	0	870	1, 2	650	0, 1	980	835
14	40B	0.405	0.27	0.75	0	0	0	0	0	0	0	0	0	840	1	550	1	785	635
15	45B	0.455	0.27	0.75	0	0	0	0	0	0	0	0	0	840	1	550	1	835	685
16	50B	0.51	0.27	0.75	0	0	0	0	0	0	0	0	0	840	2	600	0	785	540
17	40MnB	0.405	0.27	1.25	0	0	0	0	0	0	0	0	0	850	2	500	1, 2	980	785
18	45MnB	0.455	0.27	1.25	0	0	0	0	0	0	0	0	0	840	2	500	1, 2	1030	835
19	20MnMoB	0.19	0.27	1.05	0	0.25	0	0	0	0	0	0	0	880	2	2000	0, 2	1080	885
20	15MnVB	0.15	0.27	1.4	0	0	0	0	0	0.095	0	0	0	860	2	200	0, 1	885	635
21	20MnVB	0.2	0.27	1.4	0	0	0	0	0	0.095	0	0	0	860	2	200	0, 1	1080	885
22	40MnVB	0.405	0.27	1.25	0	0	0	0	0	0.075	0	0	0	850	2	520	1, 2	980	785
23	20MnTiB	0.205	0.27	1.45	0	0	0	0	0	0.07	0	0	0	860	2	200	0, 1	1130	930
24	25MnTiBRE	0.25	0.325	1.45	0	0	0	0	0	0.07	0	0	0	860	2	200	0, 1	1380	0
25	15Cr	0.15	0.27	0.55	0.85	0	0	0	0	0	0	0	0	880	1, 2	200	0, 1	735	490
26	38CrSi	0.39	1.15	0.45	1.45	0	0	0	0	0	0	0	0	900	3	600	1, 2	980	835
27	12CrMo	0.115	0.27	0.55	0.475	0	0	0	0	0	0	0	0	900	0	650	0	410	265
28	15CrMo	0.15	0.27	0.55	0.475	0	0	0	0	0	0	0	0	900	0	650	0	440	295
29	20CrMo	0.205	0.27	0.55	0.2	0	0	0	0	0	0	0	0	880	1, 2	500	1, 2	885	685
30	30CrMo	0.3	0.27	0.55	0.2	0	0	0	0	0	0	0	0	880	1, 2	540	1, 2	930	785
31	30CrMoA	0.295	0.27	0.55	0.2	0	0	0	0	0	0	0	0	880	2	540	1, 2	930	735
32	35CrMo	0.36	0.27	0.55	0.2	0	0	0	0	0	0	0	0	850	2	550	1, 2	980	835
33	42CrMo	0.415	0.27	0.65	0.2	0	0	0	0	0	0	0	0	850	2	560	1, 2	1080	930
34	12CrMoV	0.115	0.27	0.55	0.45	0.3	0	0	0	0.225	0	0	0	970	1	750	0	440	225
35	35CrMoV	0.34	0.27	0.55	1.15	0.25	0	0	0	0.15	0	0	0	900	2	630	1, 2	1080	930
36	12Cr1MoV	0.115	0.27	0.55	1.05	0.275	0	0	0	0.225	0	0	0	970	0	750	0	490	245
37	25Cr2MoVA	0.255	0.27	0.55	1.65	0.3	0	0	0	0.225	0	0	0	900	2	640	0	930	785
38	25Cr2Mo1VA	0.255	0.27	0.65	2.3	1	0	0	0	0.4	0	0	0	1040	0	700	0	735	590
39	38CrMoAl	0.385	0.325	0.45	1.5	0.2	0	0	0	0	0	0	0	940	1, 2	640	1, 2	980	835
40	40CrV	0.405	0.27	0.65	0.95	0	0	0	0	0.15	0	0	0	880	2	650	1, 2	885	735
41	50CrVA	0.505	0.27	0.65	0.95	0	0	0	0	0.15	0	0	0	860	2	500	1, 2	1280	1130
42	15CrMn	0.15	0.27	1.25	0.55	0	0	0	0	0	0	0	0	880	2	200	1, 2	785	590
43	20CrMn	0.2	0.27	1.05	1.05	0	0	0	0	0	0	0	0	850	2	200	1, 2	930	735
44	40CrMn	0.41	0.27	1.05	1.05	0	0	0	0	0	0	0	0	840	2	550	1, 2	980	835
45	20CrMnSi	0.2	1.05	0.95	0.95	0	0	0	0	0	0	0	0	880	2	480	1, 2	785	635
46	25CrMnSi	0.25	1.05	0.95	0.95	0	0	0	0	0	0	0	0	880	2	480	1, 2	1080	885
47	30CrMnSi	0.305	1.05	0.95	0.95	0	0	0	0	0	0	0	0	880	2	520	1, 2	1080	885
48	30CrMnSiA	0.31	1.05	0.95	0.95	0	0	0	0	0	0	0	0	880	2	540	1, 2	1080	835
49	35CrMnSiA	0.355	1.25	0.95	1.25	0	0	0	0	0	0	0	0	950	2	230	0, 2	1620	1280
50	20CrMnMo	0.2	0.27	1.05	1.25	0	0	0	0	0	0	0	0	850	2	200	0, 1	1180	885
